# MR-guided percutaneous microwave coagulation of small breast tumors

**DOI:** 10.1186/s13244-024-01645-4

**Published:** 2024-03-18

**Authors:** Ying Ji, Yaoyao Zhuo, Ting Li, Jingge Lian, Zilin Wang, Xinyu Guo, Dexing Kong, Kangan Li

**Affiliations:** 1 Department of Radiology, Shanghai General Hospital, Shanghai Jiao Tong University School of Medicine, No. 650 New Songjiang Road, Shanghai, 201620 China; 2 Department of Radiology, Zhongshan Hospital, Fudan University School of Medicine, Shanghai, 200000 China; 3 Department of Radiology, First People’s Hospital of Changzhou, Jiangsu, 213003 China; 4 School of Mathematical Sciences, Zhejiang University, Zhejiang, 310027 China; 5 Department of Radiology, Songjiang Hospital Affiliated to Shanghai Jiao Tong University School of Medicine, Shanghai, 201600 China

**Keywords:** Breast, MR-guided, Minimally invasive, Microwave ablation

## Abstract

**Background:**

To evaluate the technical success and patient safety of magnetic resonance-guided percutaneous microwave coagulation (MR-guided PMC) for breast malignancies.

**Methods:**

From May 2018 to December 2019, 26 patients with breast tumors measuring 2 cm or less were recruited to participate in a prospective, single-institution clinical study. The primary endpoint of this study was the evaluation of treatment efficacy for each patient. Histochemical staining with α-nicotinamide adenine dinucleotide and reduced (NADH)-diaphorase was used to determine cell viability following and efficacy of PMC. The complications and self-reported sensations from all patients during and after ablation were also assessed. The technical success of the PMC procedure was defined when the area of the NADH-diaphorase negative region fully covered the hematoxylin–eosin (H&E) staining region in the tumor.

**Results:**

All patients had a complete response to ablation with no residual carcinoma on histopathological specimen. The mean energy, ablation duration, and procedure duration per tumor were 36.0 ± 4.2 kJ, 252.9 ± 30.9 S, and 104.2 ± 13.5 min, respectively. During the ablation, 14 patients underwent prolonged ablation time, and 1 patient required adjusting of the antenna position. Eleven patients had feelings of subtle heat or swelling, and 3 patients experienced slight pain. After ablation, one patient took two painkillers because of moderate pain, and no patients had postoperative oozing or other complications after PMC. Induration around the ablation area appeared in 16 patients.

**Conclusion:**

MR-guided PMC of small breast tumors is feasible and could be applied in clinical practice in the future.

**Critical relevance statement:**

MR-guided PMC of small breast tumors is feasible and could be applied in clinical practice in the future.

**Key points:**

**•** MR-guided PMC of small breast tumors is feasible.

**•** PMC was successfully performed for all patients.

**•** All patients were satisfied with the final cosmetic result.

**Graphical Abstract:**

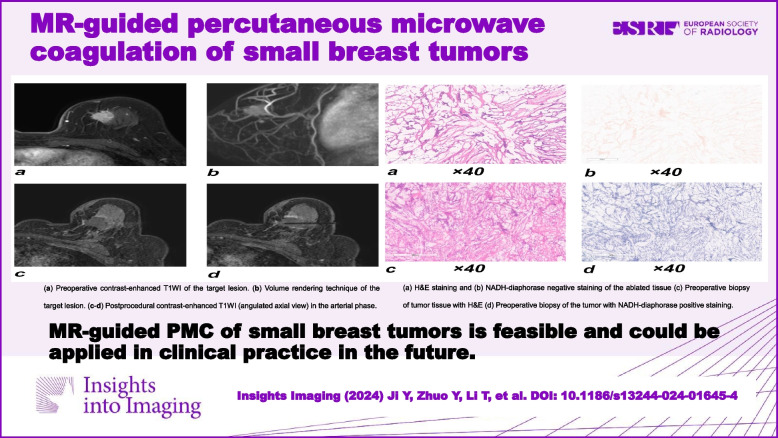

**Supplementary Information:**

The online version contains supplementary material available at 10.1186/s13244-024-01645-4.

## Background

Breast cancer is the most common malignant neoplasm among women and is associated with high morbidity and mortality [1]. The ameliorative effects and efficacy of breast surgery techniques continue to be improved [2], but surgical intervention remains inevitable, and relatively satisfactory cosmetic results are difficult to achieve.

Therefore, minimally invasive therapy for breast cancer has arisen as a fiercely debated topic in recent years [3] and has multiple advantages, including a shorter treatment time, smaller incision, less need for hospitalization, less limitation of mobility after therapy, and minimal impact on cosmetic appearance. The percutaneous treatment could be used to reduce over-treatment of screening detected, non-biologically aggressive cancers. Also, it could balance the ‘over-diagnosis’ of cancer detected with screening programs.

Percutaneous ablation techniques, such as cryoablation, radiofrequency ablation (RFA), laser ablation, microwave irradiation, high-intensity focused ultrasound (HIFU), and electroporation offered their own advantages and disadvantages in terms of success rates and degrees of ablation [4–6]. Microwave ablation produces relatively high intratumoral temperatures, resulting in larger ablation zones within a short duration and the use of one single probe [7].

Ultrasound (US) is the typical imaging modality of choice for percutaneous ablation techniques for the breast [8–10], which is affordable and easily available and has high diagnostic performance for superficial organs. However, MR performs better than US in providing information about tumor boundary and blood supply [11]. Moreover, nonsurgical ablation performed under MR guidance is more accurate in terms of soft tissue contrast, which means better identification of important tissues around the lesion, ensuring better safety and efficacy for the patient and immediate assessment of the treatment effects [12].

Some recent studies have investigated the feasibility of microwave coagulation guided by MR, mainly in liver tumors [13]. Unfortunately, no studies have yet evaluated the potential and feasibility of applying this technique to breast tumors.

Therefore, the purpose of this study was to evaluate the efficacy of MR-guided microwave coagulation in the treatment of breast carcinomas and assess the extent of ablation by comparing the biological activity of the breast tissue before and after the operation.

## Materials and methods

### Patient enrollment

Twenty-six patients in our hospital were recruited for this prospective, nonrandomized study from May 31, 2018, to December 30, 2019, with the approval of the institutional ethics committee and written informed patient consent.

The inclusion criteria were as follows: (1) core-needle biopsy-proven unifocal breast cancer by ultrasound; (2) largest breast tumor diameter of 2.0 cm or less and tumors with a lump-like appearance, unifocal as confirmed by MR enhanced imaging; (3) at least 1 cm between the tumor and the skin surface and pectoralis muscle; (4) no enlarged lymph nodes or distant metastases in ultrasonic axillary examination and computed tomography (CT) staging.

The exclusion criteria included the following (1) pregnancy or lactation; (2) the presence of more than one lesion in the breast; and (3) patient’s preference to undergo breast-conserving surgery.

Immunohistochemical analysis was performed for these patients, including hormone receptor and human epidermal growth factor receptor 2 (HER2) status, prior to percutaneous microwave coagulation (PMC) using tissue from a core-needle biopsy specimen. Meanwhile, the histological subtype of breast malignancies was also analyzed.

### Instrumentation

The microwave delivery system consisted of a microwave generator, a flexible coaxial cable, and an internal water-cooled shaft antenna. The microwave radiation frequency was 2450 MHz, and the size of the probe was 1.8 mm × 180 mm (Vison-China Medical Devices R&D Center, Nanjing, China). The output power ranged from 10 to 100 W, and the ablation pattern of microwave generator was shown in the Supplementary material (Figure S1), 40 W for 4 min was selected in this study according to previous PMC studies and our own experience [14].

**Fig. 1:**
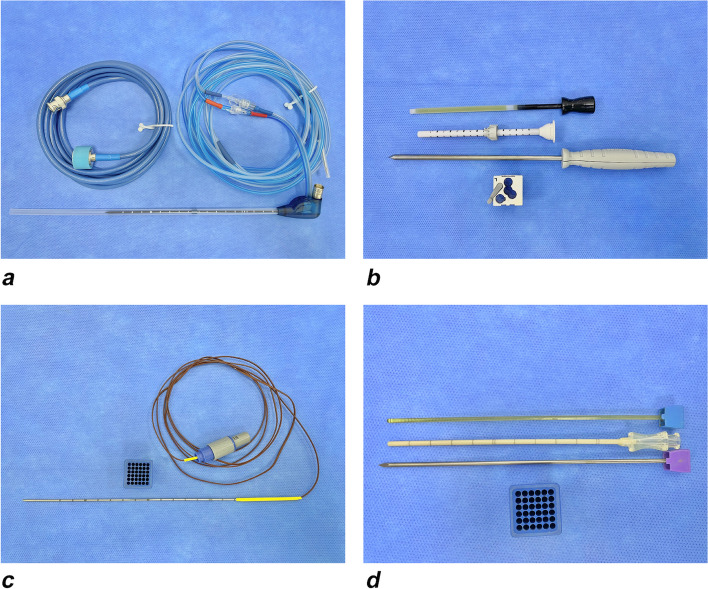
**a** Ablation setup: The disposable ablation needle is connected to a cooling water circulation system (two blue coils). **b** Needle setup for guided ablation. **c** Temperature-measuring electrode. **d** Setup for use of the temperature-measuring electrode or for preoperative biopsy

The disposable ablation needle was connected to three tubes: the microwave cable, which projected to the microwave generator, and both the entrance and exit of the water-cooling system with circulation in a 500-mL bottle of normal saline (Fig. 1a).

### MR-guided PMC

DynaCAD (Version 2.0, Invivo Corporation, FL, USA) has an extensive set of computer-aided detection (CAD) tools for performing real-time analysis and interventional procedure planning using MR imaging (MRI) patient exam data.

The grid or the post-and-pillar targeting approach was used to precisely locate the target lesion multidimensionally (horizontal distance, vertical distance, needle depth, angulation). Both the breast coil and biopsy immobilization/grid plates were properly oriented and secured on the MR scanner patient table.

Preoperative MR evaluation and real-time MR-guided percutaneous treatment of breast cancer were performed by one radiologist with over 21 years of experience in interventional breast MR. Both preoperative MR and real-time MR images were performed in the prone position in all women, using an MRI scanner (3.0 T; Achieva; Philips Healthcare) with a breast coil of 7-channel phased array. Parameters of T1WI, T2WI, and T1W contrast-enhanced sequences are in Table 1.

**Table 1:** Parameters of all sequences

	T1WI	T2WI	Contrast-enhanced
TR(ms)	596	1250	3.2
TE(ms)	8	70	1.55
NEX	1	1	/
FOV(mm)	280 × 339	280 × 340	360 × 360
Matrix size	352 × 424	352 × 423	276 × 276
Slice	32	36	29
Slice thickness(mm)	3.5	3.85	3.0
Acquisition time	1 min 28 s	1 min 30 s	6 min 49 s

The gadolinium-based contrast agent (Gd-DTPA, Magnevist; Bayer Healthcare, Germany) was injected intravenously (0.2 mmol/kg body weight at a rate of 3 mL/s), using a power injector, followed by a 20-mL saline flush, to obtain postprocedural T1W contrast-enhanced images

MR was used to identify the lesion, and an image in the maximum plane of the lesion was acquired (the long and short axes of the image can be measured as a1 and b1, respectively). All procedures were carried out in the MR examination room with the patient prone on the operation table and following the subcutaneous injection of local anesthesia (covering the subcutaneous tissue, target tumor, and surrounding tissue with 0.5–2% lidocaine, according to the lesion location with MR images), which was less likely to produce pain.

**Fig. 2:**
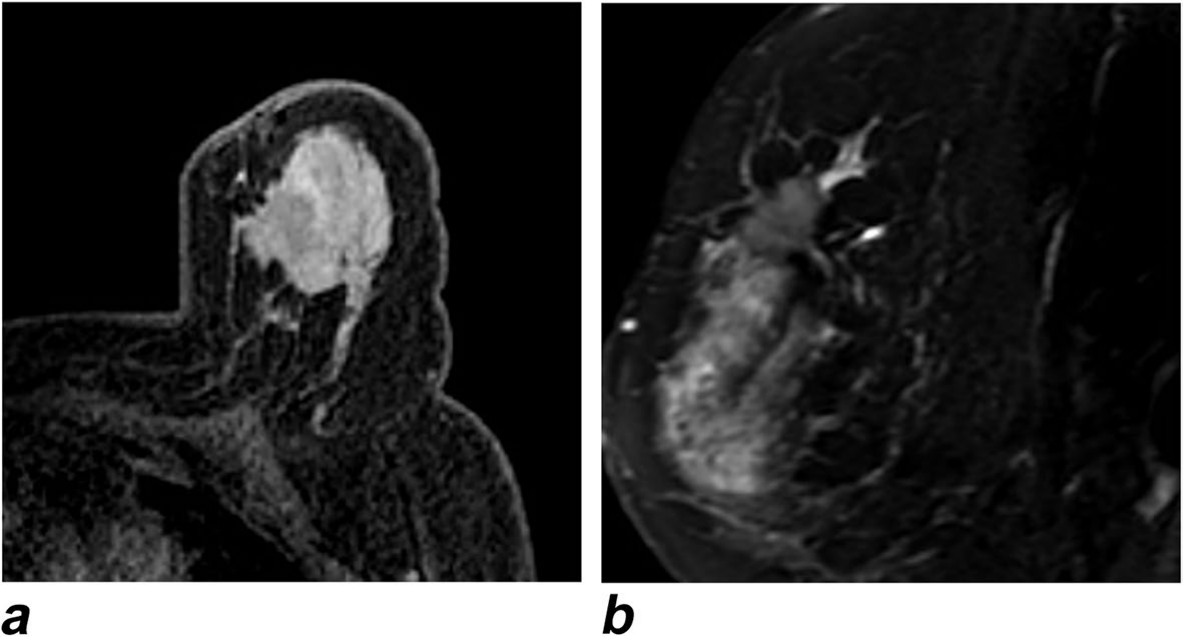
A 58-year-old woman with invasive ductal carcinoma (medial-upper quadrant, 1.7 × 1.6 cm), preablation. **a** Unenhanced T1WI of the breast lesion, angulated axial view. **b** T2WI of the breast lesion, angulated sagittal view

**Fig. 3:**
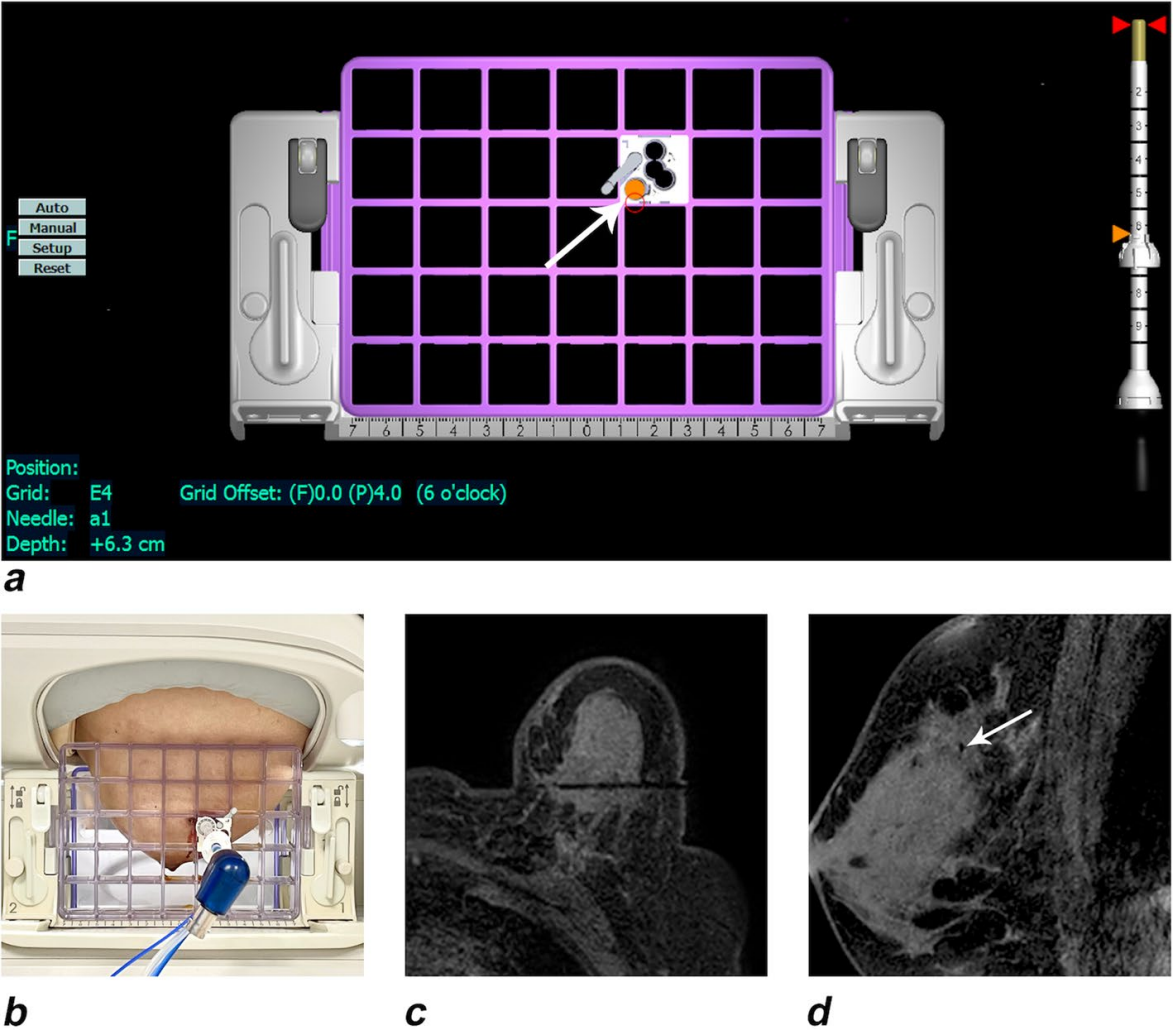
**a** The white marker indicates the entry point of the antenna in DynaCAD. **b** Locating the lesion in clinical practice according to DynaCAD software. **c** The tip of the disposable ablation needle gradually passed through the largest diameter of the lesion. **d** Sagittal view of the needle path, indicated by the white arrow

With the help of the ablation needle guide set (Fig. 1b), after precisely locating the lesion with real-time unenhanced T1-weighted (T1W) and T2-weighted (T2W) MR images (Fig. 2), the tip of the disposable ablation needle was advanced into the tumor to the opposite edge so that the microwave emission area could precisely cover the entire tumor (Fig. 3). After testing the cold-water cycling system, under real-time MR monitoring, when the ablation zone fully covered the target tumor with a sufficient safety margin of > 5 mm on MR images, the PMC process was terminated. If the ablation zone (manifesting as a hyperintense area on unenhanced T1WI and a hypointense area on T2WI) was considered insufficient, the procedure was repeated with proper adjustment such as extending the ablation time or redirecting the ablation needle.

Just when the procedure was finished, postprocedural T1W contrast-enhanced images (use contrast media only this time) were acquired to evaluate technical results and the creation of a satisfactory coagulation zone: hypointensity in the treatment zone and enhancement of nodular and circular shape (a fine enhancement) in the peripheral tissue due to congestion after treatment. A special antenna with a temperature sensor (Vison-China Medical Devices R&D Center, Nanjing, China) was used to consistently measure the temperature 5 mm from the site of radiation to minimize geothermal damage to the surrounding normal tissue (Fig. 1c, d). The method of application was similar to the placement of an ablation needle.

### Pathologic analysis

**Fig. 4:**
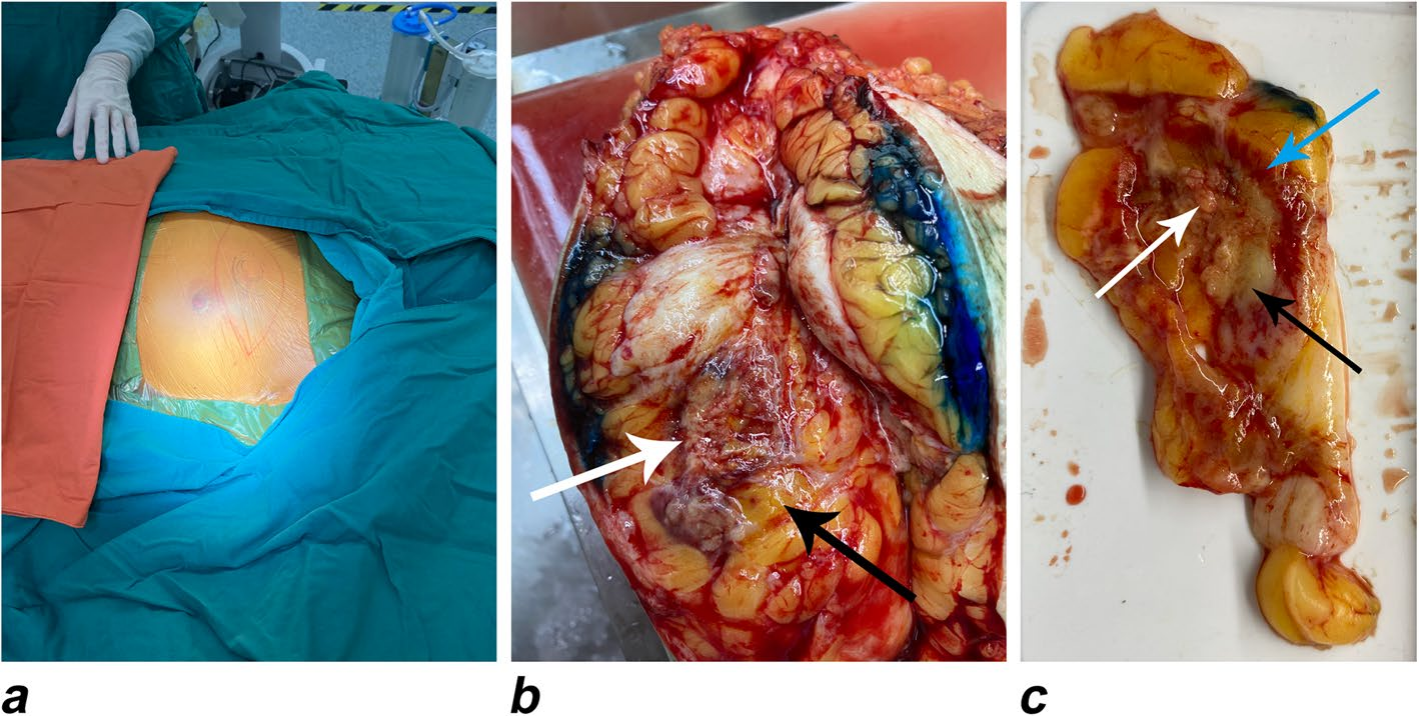
**a** Preoperative positioning and preparation. **b** Macroscopic evaluation of the treatment zone before surgical resection (white arrow = tumoral ablation zone; black arrow = breast fat around the ablation zone). **c** Macroscopic evaluation of the treatment zone on the specimen (white arrow = tumoral necrosis zone; black arrow = ablation zone of peritumoral breast fat, approximately 0.5–1.0 cm in size). The blue arrow indicates the hyperemic ring around the ablated area

After PMC, each patient was sent to undergo prescheduled mastectomy (Fig. 4a) to obtain pathological specimens, which were used to confirm the complete loss of tumor biological activity. After surgery, the specimen was cut open along the needle tract (Fig. 4b), and two sections were taken to obtain the full maximum cross-section of the ablated tumor (Fig. 4c). In addition to standard histological assessment with hematoxylin–eosin (H&E) staining, nicotinamide adenine dinucleotide, reduced (NADH)-diaphorase staining of the tumor tissue was performed to assess tumor viability; staining of the breast tumor tissue obtained by preoperative biopsy was also performed for comparison. First, one section of the breast tissue specimen was fixed in formalin and embedded in paraffin. The other section was snap-frozen in liquid nitrogen (- 80 °C) and stained with NADH-diaphorase. Tumor cells that lost their vitality due to ablation would not retain staining with a dark blue color; however, their outlines could still be depicted on H&E staining. The long and short axes of the tumor in the H&E staining and NADH-diaphorase negative staining regions were defined as a2 and b2 and a3 and b3, respectively. The formula *π* × *A* × *B*/4 was used to calculate the tumor area of each slice; if the area of the NADH-diaphorase-negative region fully covered the H&E staining region in the tumor, the ablation was considered complete. Breast tumor tissue obtained by preoperative biopsy was used to demonstrate the reliability of pathological staining. The complications and self-reported sensations of all patients during and after percutaneous treatment were also assessed. After the endpoint of evaluation after breast surgery, sentinel lymph node biopsy (SLNB) was performed with ultrasound guidance and the surgeon carried out corresponding treatment.

### Statistical analysis

Numerical data are reported as the mean ± standard deviation. The Wilcoxon rank-sum test was performed to assess the difference between the maximum area of the tumor in H&E-stained sections and on MRI, and the paired *t* test was used to assess the difference between the tumor area in H&E and NADH-diaphorase negative staining area. All statistical analyses were performed by using SPSS 22.0 software, and *p* < 0.05 was considered to signify statistical significance.

## Results

### Patient and clinical data

Between 2018 and 2019, 26 women were enrolled in this study. The descriptive statistics are shown in Table 2.

**Table 2:** Descriptive statistics for all 26 patients

Age (years)	52.0 ± 12.2 (range 31–75)
Location	
Left	17
Right	9
MR	
a1 (cm)	1.488 ± 0.255 (range 1.1–1.9)
b1 (cm)	1.219 ± 0.325 (range 0.5–1.9)
ER positive	23
PR positive	17
HER2 positive	6
High expression of Ki67	13
Molecular subtype	
Luminal A	12
Luminal B (HER2 negative)	6
Luminal B (HER2 positive)	5
HER2 enriched (nonluminal)	1
Triple negative	2

Except for age and MR, the data are the number of patients

Luminal A: estrogen receptor (ER) and progesterone receptor (PR) positive, Ki67 level less than 20%, HER2 negative. Luminal B (HER2 negative): ER positive and HER2 negative (PR < 20% or Ki67 ≥ 20%). Luminal B (HER2 positive): ER and HER2 positive (PR < 20% or Ki67 ≥ 20%). HER2 enriched (nonluminal): ER and PR negative and HER2 positive. Triple negative: ER, PR, and HER2 negative

### Treatment procedure and complications

**Fig. 5:**
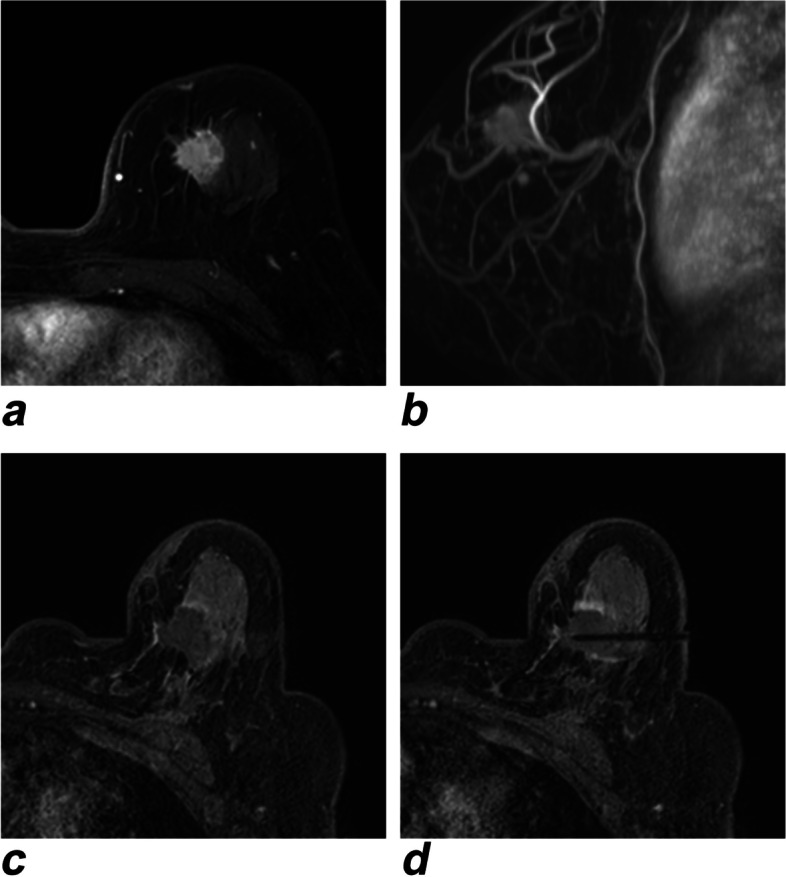
A 58-year-old woman. **a** Preoperative contrast-enhanced T1WI of the target lesion. **b** Volume rendering technique (VRT) reconstruction of the target lesion. **c**, **d** Postprocedural contrast-enhanced T1WI (angulated axial view) showing hypointensity in the treatment zone and peripheral enhancement of circular and nodular shape in the arterial phase

The PMC procedure was performed smoothly with local anesthesia for all patients (Table 3). All patients underwent complete ablation of their breast tumors, 14 patients required prolonging ablation time due to insufficient safety margins under real-time MR monitoring, while another patient required adjusting the position of the antenna to enlarge the ablation zone to fully cover a relatively large breast tumor of irregular shape during the operation. All T1-enhanced images obtained immediately after percutaneous treatment showed complete ablation of the mass (Fig. 5). Three patients experienced slight pain during the ablation process, and an appropriate amount of lidocaine mixed with saline was administered to the patients in a timely manner. All patients were satisfied with the final cosmetic result after PMC, which consisted of one or two pinhole marks that were covered by a needle eye sticker and waterproof applicator so that the daily lives of the patients would not be affected at home. Two days later, the sticker and applicator could be removed.

**Table 3:** Comparison and evaluation pertaining to the pre- and postablation periods

H&E	
a2 (cm)	1.154 ± 0.318 (0.5–1.5)
b2 (cm)	0.977 ± 0.260 (0.5–1.5)
NADH-diaphorase	
a3 (cm)	1.650 ± 0.280 (1.0–2.0)
b3 (cm)	1.412 ± 0.234 (0.9–1.8)
Energy (W)	35.962 ± 4.247 (30–45)
Ablation time (s)	252.923 ± 30.857 (200–310)
Procedure time (min)	104.231 ± 13.468 (90–130)
Prolonged ablation	14
Antenna repositioned	1
Pain during the ablation	3
Feelings of subtle heat or swellingduring the ablation	11
Satisfaction with the final cosmetic result	26
Took painkiller(s) after the ablation	1
Postoperative oozing	0
Induration around the ablation area	16

Only one patient took two painkillers (Ibuprofen, 0.2–0.4 g at a time, every 6 to 4 h) because of moderate pain within 2 days after PMC. No patient had postoperative oozing or other complications, such as breast inflammation or skin burns, after PMC. Induration owing to swelling and fibrosis around the ablation area appeared in 16 patients but was psychologically accepted by all of them. Eleven patients had feelings of subtle heat or swelling during the ablation, but it did not cause them any significant discomfort.

### Pathologic results

**Fig. 6:**
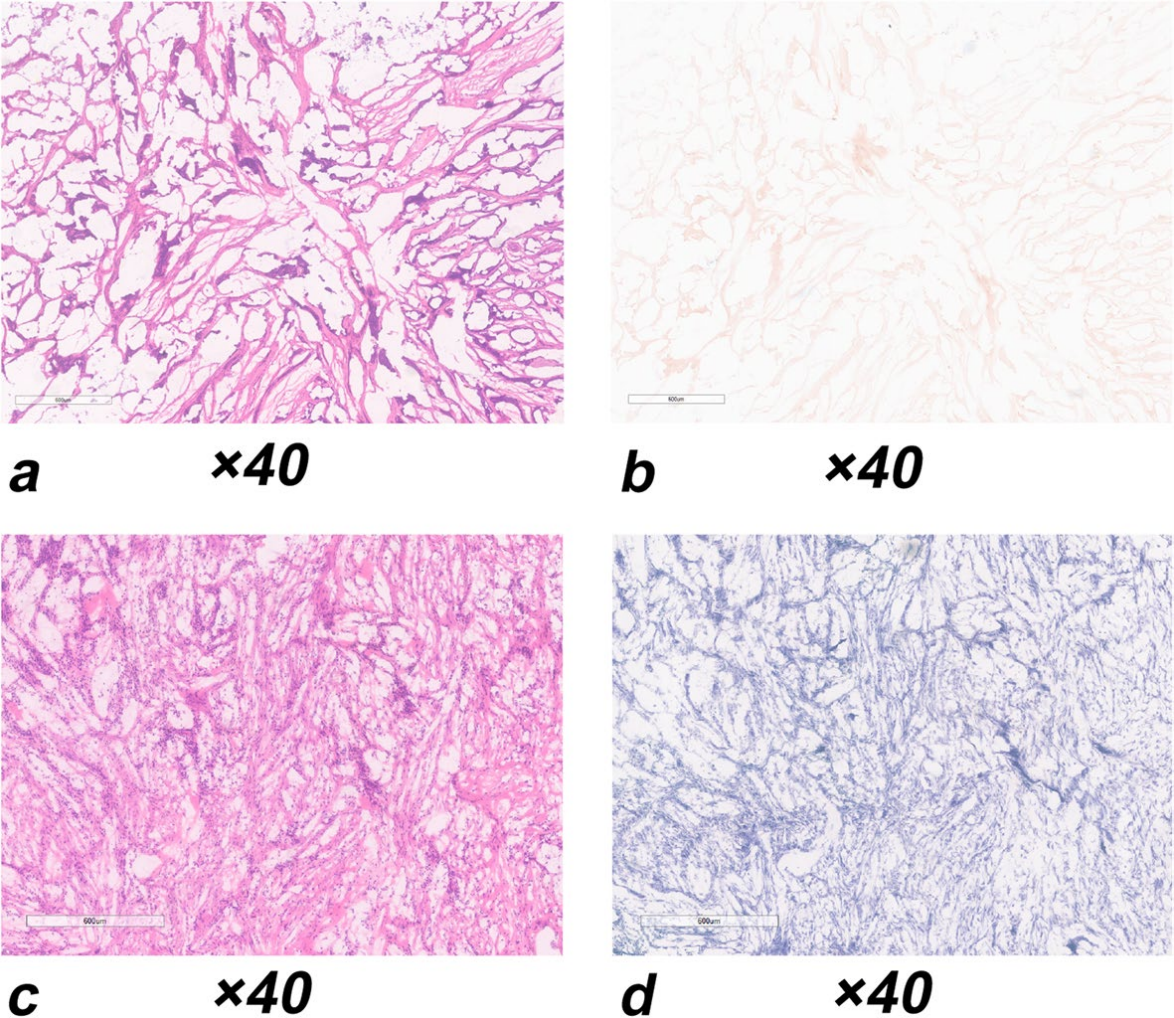
A 58-year-old woman with invasive carcinoma. Confirmation of the efficacy of PMC therapy means the area of the NADH-diaphorase negative staining fully covered the H&E staining region in the tumor. **a** H&E staining and **b** NADH-diaphorase negative staining reveal that the structure of the ablated tissue is unchanged; no tumor disintegration is observed. **c** Preoperative biopsy of tumor tissue with H&E staining. **d** Preoperative biopsy of the tumor tissue with NADH-diaphorase positive staining

Histopathologically, 26 patients were diagnosed with malignant breast lesions, including 21 invasive carcinoma (20 invasive ductal carcinoma and 1 invasive lobular carcinoma), 1 solid papillary carcinoma, 2 mucinous carcinoma, and 2 ductal carcinoma in situ (DCIS). The structure of the ablated tissue and the shape of tumor cells were intact and appeared unchanged in H&E staining, reflecting the size of the tumor at the pathological level. The extent of breast cancer was significantly greater on both MRI and NADH-diaphorase negative staining than on H&E staining (1.519 vs. 0.836 mm^2^, *p* < 0.001; 1.872 vs. 0.934 mm^2^, *p* < 0.001). All tumors were confirmed to have undergone complete ablation after PMC, with their shape intact (Fig. 6). All data pertaining to the pre- and postablation periods are shown in Table 3.

## Discussion

In this report, percutaneous MR-guided breast tumor ablation was performed with a technical success rate of 100% for all 26 patients with trivial complications, which demonstrates that this procedure is indeed feasible.

The final histopathology was the gold standard for detecting residual lesions. In other words, the real weakness of image-guided breast cancer ablation techniques was the lack of pathological examination of surgical specimens, which was precisely one of the highlights of this article.

A successful ablation technique depends on the accurate insertion of the antenna into the target lesion, real-time monitoring, and adjustment of the treatment as needed, and subsequent evaluation of the success of the technique.

The benefits of microwave ablation include higher constant intratumoral temperatures and faster ablation times than those of RFA in breast tumors [7]. In our study, the mean ablation time was 4.2 min. Moreover, microwaves do not affect the MR images because they generally operate at a frequency between 915 MHz and 2.45 GHz [13]. Jie Yu et al. also suggested that microwave ablation achieved similar short-term results in breast cancer control compared with nipple-sparing mastectomy [15]. In addition, a recent study by Zhou suggested that microwave ablation may be not only a prominent local therapy for breast cancer, but also an inducer of antitumor immunity (Th1-type immune response and CD4 + T_EM_ response)—exceedingly beneficial for the long-term prognosis of the patient [16].

Correct size assessment is a crucial condition for complete ablation of the breast tumor. If the microscopic extent of the tumor is inappropriately underestimated, the risk of breast cancer recurrence would be increased due to the presence of tumor remnants. Berg suggested that US was likely to underestimate the extent of breast tumors [17], while in our study, the extent of the breast tumors on MRI was statistically larger than that with pathology (*p* < 0.001), which indicated that MR was preferably chosen to observe and verify the extent of the tumor. In breast cancer ablation, MR image guidance was mainly used for HIFU so far [18, 19]. Therefore, this paper innovatively applied it to microwave ablation. According to our findings, MR guided PMC was indeed suitable for patients recruited in our study.

In a study by Zhou et al. [20], tumors of two patients were unsuccessfully ablated with microwave coagulation due to poor positioning of the antenna. Undoubtedly, the proper fixation of the antenna position was a critical step in the ablation process [21]. Stereotactic approaches under MRI and monitored throughout the whole procedure helped fixing the position of the antenna (Fig. 3). This is challenging during US-guided ablation due to its reliance on the technical experience of the operator and the difficulty in guaranteeing the ablation needle remains unmoved during the process. The extents of the tumors were precisely evaluated, and the specific signal differences between the coagulated and noncoagulated tissue were clearly displayed on the MR images of all patients with breast tumors [22]. These factors all reduced the possibility of incomplete ablation or the development of adverse events due to poor tissue contrast or certain complex anatomical locations relative to the US-guided procedure.

In another study by Zhou [23], some patients were unable to tolerate the pain of the full treatment duration of microwave ablation. The interruption of the treatment procedure most likely affected the final treatment result. In our study, three patients felt slight pain during ablation, but the operation did not cease due to the timely supply of local anesthetic; no other adverse events were noted during PMC. This is a boon for patients who could not safely undergo anesthetic for breast surgery. After the treatment, one patient took painkillers because of moderate pain, and no other complications, such as bleeding or infection, were observed. Induration owing to swelling and fibrosis around the ablation area appeared in 16 patients but was psychologically accepted by all of them and thus was not considered an adverse event. In general, the patients tended to have satisfactory intraoperative and postoperative experiences.

We did not encounter any thermal injury to the skin or pectoralis muscle, which is a low but potential risk of PMC, probably related to the inclusion criteria [4] (criteria 2 and 3 [24, 25]). In addition, real‐time monitoring of conditions such as thermal changes [26], excellent control of the trajectory of the antenna, and instant potential adjustment of the coagulation procedures aided in optimizing the safety of PMC. Postoperative breast infection did not occur in this study. Manenti et al. [27] suggested administering oral prophylactic antibiotic therapy 5 days after the procedure, which could theoretically be ideal for infection prevention.

MRI is also the most suitable choice for posttreatment evaluation [12, 15], which is as critical as an accurate preoperative assessment of the tumor [28]. MRI has been used as the gold standard for post-RFA treatment assessment for breast cancer in many studies [29]. With MR-guided PMC, a postablation scan can be immediately attained to ensure complete ablation.

With US-guided ablation, the formation of gas bubbles during the treatment process leads to reflection and scattering of US waves, which can disguise the actual coagulation zone and interfere with effective US imaging to some extent [29–31]. Therefore, the margin of the ablation area, and changes in the surrounding structure, was sometimes hazy making it difficult to evaluate the ablation progress. In contrast, MRI is able to continuously visualize the vessels without the need for intravenous contrast, and subtle signal changes in the surrounding tissue can be more effectively displayed [32]. In our study, the immediate postoperative enhanced T1W images clearly reflected the extent of mass necrosis and the changing signals of the surrounding tissue, which provided opportunities for timely remedy for cases with an insufficient ablation range.

There are some limitations to our study. First, we did not compare different image guidance modalities and ablation techniques. Moreover, the investigated patient cohort was relatively small, and no information was provided on other imaging techniques like mammography and ultrasound. In addition, a lack of long-term follow-up and SLNB make it difficult to obtain information on local tumor recurrence and axillary management. Finally, the long duration coupled with the high costs of the operation are the main reasons for the limited application of MR-guided PMC in breast tumors.

## Conclusion

In conclusion, MR-guided PMC is a safe and accurate, minimally invasive therapeutic approach for breast malignancies due to its precision targeting, ability to be monitored in real time, and ability to immediately evaluate success. If possible, more research should focus on long-term follow-up after MR-guided PMC obtaining information on local tumor control and recurrence, expanding the study cohort, further reducing operation time and expenses. If so, this advanced technique is hopeful to be universally introduced to actual clinical practice as an alternative treatment for traditional surgical procedures for breast cancer.

### Supplementary Information

**Supplementary Material 1.**

## Data Availability

The datasets generated during and/or analyzed during the current study are not publicly available.

## Abbreviations

CAD: Computer-aided detection
ER: Estrogen receptor
H&E: Hematoxylin-eosin
HER2: Human epidermal growth factor receptor 2
MRI: Magnetic resonance imaging
NADH: α-Nicotinamide adenine dinucleotide, reduced
PMC: Percutaneous microwave coagulation
PR: Progesterone receptor
RFA: Radiofrequency ablation
T1W: T1-weighted
T2W: T2-weighted
US: Ultrasound
