# Supplementary material for: MR-guided percutaneous microwave coagulation of small breast tumors
Source: Insights Imaging. 2024 Mar 18;15:76. doi: 10.1186/s13244-024-01645-4 (PMC10948645; doi:10.1186/s13244-024-01645-4)
Supplement: Supplementary file 1 — Supplementary Material 1. [file 13244_2024_1645_MOESM1_ESM.pdf]

MR-guided percutaneous microwave coagulation of small breast tumors

ELECTRONIC SUPPLEMENTARY MATERIAL

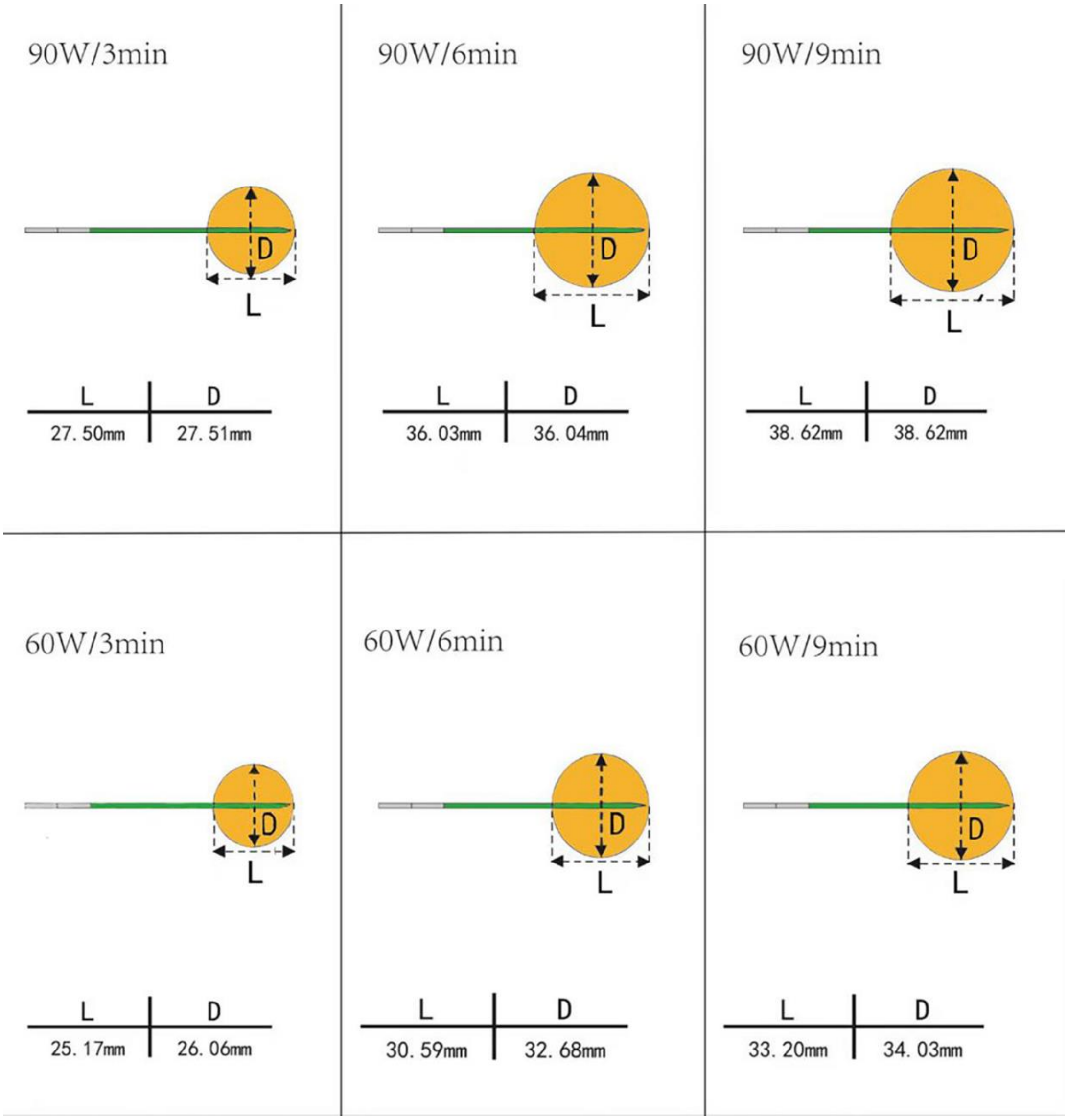

Figure S1: The ablation pattern of microwave ablation with different power and duration.
